# Double Parasitism by Two Cuckoo Gentes in a Daurian Redstart Nest

**DOI:** 10.1002/ece3.73968

**Published:** 2026-07-02

**Authors:** Jinggang Zhang, Peter Santema, Chenyang Zhao, Zixuan Lin, Jianqiang Li, Wenhong Deng, Bart Kempenaers

**Affiliations:** ^1^ Department of Biology, Faculty of Arts and Sciences Beijing Normal University Zhuhai China; ^2^ Ministry of Education Key Laboratory for Biodiversity Sciences and Ecological Engineering, College of Life Sciences Beijing Normal University Beijing China; ^3^ Department of Ornithology Max Planck Institute for Biological Intelligence Seewiesen Germany; ^4^ School of Ecology and Nature Conservation Beijing Forestry University Beijing China

**Keywords:** brood parasitism, common cuckoo, Daurian redstart, multiple parasitism

## Abstract

Brood parasites lay their eggs in the nest of other species, thereby transferring the costs of parental care to their hosts. Occasionally, more than one female lays an egg in the same host nest, a phenomenon known as multiple parasitism. In the common cuckoo *Cuculus canorus* , multiple parasitism occurs regularly in some host systems with high cuckoo densities, although its frequency varies substantially among host species and populations. Here, we report a case of double parasitism in the nest of a forest‐breeding host, the Daurian redstart *Phoenicurus auroreus* . Two cuckoo gentes occur at our study site: one lays blue eggs resembling those of redstarts (“redstart‐cuckoo”), whereas the other lays grey eggs resembling those of white wagtails *Motacilla alba* (“wagtail‐cuckoo”). Of 182 parasitized redstart nests recorded between 2018 and 2024, 180 were by a redstart‐cuckoo and only two were by a wagtail‐cuckoo. In the focal doubly parasitized nest, first a redstart‐cuckoo egg appeared, followed 4 days later by a wagtail‐cuckoo egg. Despite parasitism by redstart‐cuckoos being much more common, we never found a nest with two redstart‐cuckoo eggs. These observations raise the possibility that interactions among cuckoo females, including potential differences in tolerance or spatial overlap between gentes, may influence the occurrence of multiple parasitism. We therefore hypothesize that female cuckoos may be more tolerant towards females of other gentes, or less effective at excluding them, although this interpretation remains speculative.

## Graphical abstract

Double parasitism is rare in forest‐breeding hosts of the common cuckoo, where females typically defend exclusive territories. We report a unique case in a Daurian redstart nest involving two different cuckoo gentes, despite one gens overwhelmingly dominating parasitism events in the population. This observation suggests that female cuckoos may be less effective at excluding, or more tolerant of, intrusions by females of different gentes, highlighting a previously understudied aspect of inter‐gens interactions.

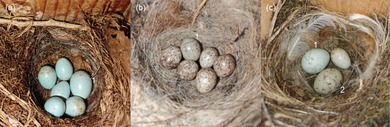

## 1 Introduction

Obligate avian brood parasites lay their eggs in the nests of other species, thereby transferring the costs of parental care to their hosts (Davies 2000). This parasitic behaviour selects for defensive adaptations in host species which, in turn, leads to counteradaptations in the parasitic species (Davies 2000). The evolutionary arms‐race between avian brood parasites and their hosts is a classic model for the study of coevolution (Soler 2014). Brood parasites may also compete with each other, especially when suitable host nests are limited. One outcome of such competition is that more than one parasitic female may lay an egg in the same host nest (Andersson and Åhlund 2012), a phenomenon known as multiple parasitism (Soler 2025).

Multiple parasitism occurs regularly in non‐evicting brood parasites, in which chicks do not actively evict nestmates (Rothstein 1990). For example, both multiple parasitism and repeated parasitism (where multiple parasitic eggs were laid by the same female) are common in the brown‐headed cowbird *Molothrus ater* (Rivers et al. 2012), shiny cowbird *M. bonariensis* (de la Colina et al. 2016), and screaming cowbird *M. rufoaxillaris* (Ursino et al. 2020). Although multiple parasitic chicks can be raised in the same nest in such non‐evicting species, it may lead to reduced survival chances for each parasitic chick (Goguen et al. 2011). A notable exception is the great spotted cuckoo *Clamator glandarius*, in which multiple parasitism is common and appears to result in increased fledging success of the parasitic chicks (Soler et al. 2023). By contrast, multiple parasitism is much rarer in evicting parasites. In these species, the first‐hatched parasitic chick evicts all nestmates and unhatched eggs, so that only a single parasitic chick can ultimately fledge from a multiply parasitized nest (Davies 2000).

The common cuckoo *Cuculus canorus* (hereafter “cuckoo”) is the most intensively studied of the evicting brood parasites (Davies 2000; Schulze‐Hagen et al. 2009). Multiple parasitism occurs regularly in some cuckoo host species. For instance, > 30% of parasitized nests in a population of great reed warblers *Acrocephalus arundinaceus* contained more than one cuckoo egg (Moskát and Honza 2002; Honza et al. 2022). Frequent multiple parasitism has also been reported in other great reed warbler populations (Zölei et al. 2015; Marton 2021) and in other host species, such as oriental reed warblers *A. orientalis* (Yang et al. 2014; Li et al. 2016), Eurasian reed warblers *A. scirpaceus* (Wyllie 1981), and Azure‐winged magpies *Cyanopica Cyana* (Yamagishi and Fujioka 1986). A shared feature of these host species is that they breed in habitats with a high density of cuckoos (Soler 2025).

Cuckoo females belong to one of numerous host‐specific races, known as ‘gentes’ (singular: ‘gens’), each producing eggs that mimic those of their respective host species (Brooke and Davies 1988; Moksnes and Røskaft 1995; Merondun et al. 2025). Females belonging to the same gens target the same host species, and individual females typically specialize on a single host species (Koleček et al. 2021), potentially leading to more intense competition among females for suitable host nests. In contrast, females from different gentes do not target the same host species, and competition for nests should therefore be weak. Thus, a female cuckoo may show greater tolerance towards females of a different gens compared to females of the same gens when multiple gentes co‐occur and females can recognize them (Nakamura and Miyazawa 1997), especially when territory defence is costly or energetically demanding (Ord 2021). However, whether cuckoo females can discriminate among gentes remains unclear, and the cues underlying such recognition—if present—are unknown.

**FIGURE 1:**
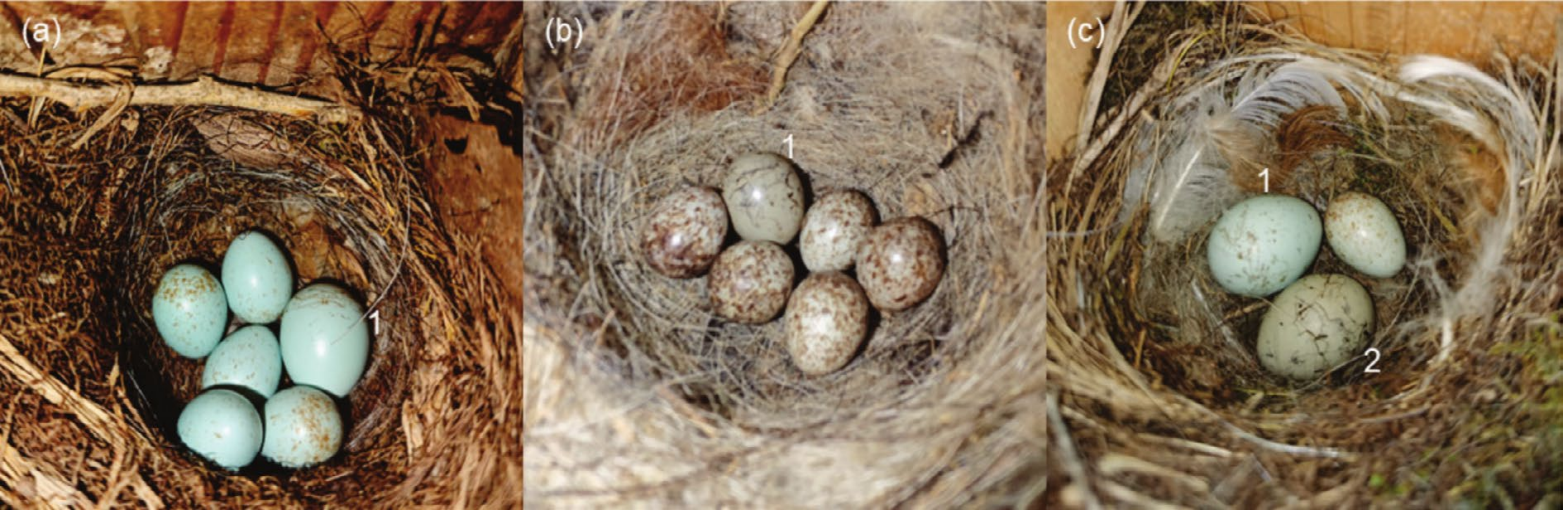
(a) A blue‐egg Daurian redstart nest containing a common cuckoo egg. (b) A white wagtail nest containing a cuckoo egg. (c) A blue‐egg Daurian redstart nest containing two cuckoo eggs of different gentes. All cuckoo eggs are numbered.

Here we report a rare case of double parasitism in a nest of Daurian redstarts *Phoenicurus auroreus* (hereafter, “redstart”), a common host of the cuckoo that breeds in forests and urban habitats. Redstarts exhibit a distinct egg‐colour dimorphism, with some females laying blue eggs and others laying pink eggs (Zhang et al. 2021). At our study site in northeast China, two cuckoo gentes occur sympatrically. One lays pale blue eggs resembling the blue morph of redstart eggs and typically parasitises redstart nests (hereafter “redstart‐cuckoo”, Figure 1a). The other gens lays grey eggs with fine markings resembling those of the white wagtail *Motacilla alba* and typically parasitises white wagtail nests (hereafter “wagtail‐cuckoo”, Figure 1b). This situation provides a unique opportunity to document interactions between cuckoo females belonging to different gentes.

## 2 Methods

Our study was conducted in ShuangYu, a village in northeastern China (43°37′19″ N and 126°09′54″ E). The village is about 300 ha and surrounded by arable fields and secondary forest. Daurian redstarts breed at high density in and around the village. They typically build nests in concealed sites, including cavities in trees, rocks, walls and eaves, but also readily use artificial nest boxes (Zhang et al. 2021). Between 2016 and 2024, we installed and maintained 240 nest boxes in the study area, with adjacent boxes spaced approximately 50 m apart. Since 2018, we have monitored Daurian redstarts' reproductive behaviour throughout the breeding season.

Redstarts in our study site typically produce two clutches per breeding season, with peak egg‐laying occurring in late April and early June (clutch size (mean ± SD): 6.4 ± 0.6 (*n* = 99) and 5.5 ± 1.0 (*n* = 163) in the first and second clutch, respectively; Zhang et al. 2021). Cuckoos generally arrived at the breeding grounds around mid‐May, by which time most redstart nests were either in late incubation or at the nestling stage of their first clutch, making them unsuitable for parasitism (Zhang et al. 2021). Therefore, parasitism occurred only during the second egg‐laying period, with an overall rate of 14.3% (Zhang et al. 2023). During each second egg‐laying period, we monitored approximately 200 nests annually (range 118–265), of which more than half were located in nest boxes. We checked nests at least once per day during egg laying and early incubation to monitor parasitism. Based on variation in cuckoo egg phenotypes (background colour, pattern, and size; Šulc et al. 2022), and temporal patterns of egg laying, we infer the presence of multiple females in our study area. For example, multiple newly laid cuckoo eggs were occasionally found on the same day (e.g., three eggs on 10 June 2019 and 19 June 2024, and two eggs on 20 June 2019, and 15 June, 25 June, and 8 July 2024), whereas a single female cuckoo is generally assumed to lay only one egg every 2 days. Together, these observations suggest that approximately 10–15 cuckoo females may have used our study area every year (Zhang et al. 2023).

## 3 Results

On 23 June 2024, we found a cuckoo egg and two blue host eggs in a redstart nest (Nest ID: 24034; laying date: 19 June; clutch size: 3; incubation started on 21 June). The cuckoo egg was pale blue, typical for redstart‐cuckoos. When we checked the nest on 27 June, we discovered a second cuckoo egg along with the first cuckoo egg and one remaining host egg (Figure 1c). This second cuckoo egg was grey with fine markings, typical for wagtail‐cuckoos. When we inspected the nest the following day, the wagtail‐cuckoo egg had been ejected, presumably by the host female.

Over a 7‐year period (2018 to 2024), we recorded a total of 182 parasitism events in redstart nests. Of these, 180 nests were parasitized by a redstart‐cuckoo, whereas only two were parasitized by a wagtail‐cuckoo. Thus, in redstart nests, parasitism by redstart‐cuckoos was overwhelmingly more common than parasitism by wagtail‐cuckoos. The fact that the only case of double parasitism ever reported in our population involves a wagtail‐cuckoo is thus notable and requires explanation.

## 4 Discussion

Among 182 parasitized redstart nests monitored over 7 years, we found only a single case of multiple parasitism (< 1%). We did not individually mark cuckoo eggs at laying; therefore, we cannot exclude the possibility that previously laid cuckoo eggs were removed by subsequent cuckoo females, which may lead to underestimation of multiple parasitism (Šulc et al. 2016). In addition, rapid host ejection of parasitic eggs before nest inspections could also result in some cases of multiple parasitism going undetected. Interestingly, frequent multiple parasitism has been reported in common redstarts *P. phoenicurus* , where 41 cases of multiple parasitism were observed among 353 parasitized nests over 16 years (11.6%) (Thomson et al. 2016; Abaurrea et al. 2025). A plausible explanation for the discrepancy in multiple parasitism frequency between these two closely related hosts is the difference in overall parasitism rate. The parasitism rate in our population (15.6%) is substantially lower than that reported for common redstarts (33.0%) (Thomson et al. 2016; Abaurrea et al. 2025). In great reed warblers, the annual rate of multiple parasitism is also positively associated with the annual parasitism rate (Honza et al. 2022). Together, these findings suggest that the rarity of multiple parasitism in Daurian redstarts may result from low cuckoo density.

The expected probability of double parasitism based on the overall parasitism rate in our population (14.3%) is approximately 2.0% (14.3% × 14.3%), which would correspond to about 26 double‐parasitized nests. This estimate is considerably higher than the single case observed. By contrast, in great reed warblers, the expected frequency based on the overall parasitism rate (54.3%) closely matches empirical observations (Honza et al. 2022). Such a close match appears to occur in multiple other host species, such as common redstarts (Abaurrea et al. 2025), large‐billed gerygones *Gerygone magnirostris* , jungle babblers *Argya striata*, and northern mockingbirds *Mimus polyglottos* (Soler 2025). However, this comparison should be interpreted with considerable caution because the calculation of the expected probability of double parasitism assumes that multiple cuckoo females have access to the same host nests and that already parasitized nests have the same probability of being parasitized again. Neither assumption is likely to be met in our study system. Based on observed geographical patterns of cuckoo egg appearance, female cuckoos in our study area seem to have non‐overlapping territories (within separate areas, cuckoo eggs look alike), and a cuckoo female does not lay multiple eggs in the same nest (Honza et al. 2022). Nevertheless, our study suggests that factors limiting access to nests (e.g., low density or territoriality of cuckoo females) may contribute to the rarity of multiple parasitism in Daurian redstarts.

Notably, the only case of double parasitism recorded in our population involved eggs from two different cuckoo gentes that locally occur sympatrically: the redstart‐cuckoo and the wagtail‐cuckoo. Based on the relative frequencies of both egg types in redstart nests, it would have been much more likely that the second egg in the doubly parasitized nest would be from a redstart‐cuckoo. While anecdotal, the fact that the only case of double parasitism in our population involved eggs of two different cuckoo gentes raises questions about interactions among females of different gentes.

We hypothesize that cuckoo females may be either less effective at defending their territory against intrusions by females of other gentes than by females of their own gens, or more tolerant towards females of other gentes because they usually do not compete for the same nests. During the breeding season, cuckoo females generally respond aggressively towards intrusions of conspecific females into their territory (Moskát and Hauber 2019; Moskát et al. 2020). This hypothesis relies on the assumption that cuckoo females can recognize the gens identity of intruding individuals; however, such discrimination has not yet been demonstrated. Moreover, the cues underlying such recognition—whether visual, behavioural, or otherwise—remain unclear, rendering this mechanism speculative. An alternative explanation is that females of different gentes encounter each other less frequently. Behavioural differences, such as variation in microhabitat use, host‐searching strategies, or daily activity patterns, may reduce the likelihood of interactions between females of different gentes. Under this scenario, reduced inter‐gens competition arises passively from ecological segregation rather than active discrimination. However, these interpretations remain speculative because we lack direct behavioural observations of interactions among cuckoo females, and females of different gentes cannot currently be distinguished reliably based on their visual appearance. Moreover, it remains unclear whether (and how) females can discriminate between conspecific females of different gentes. Future studies examining spatial and temporal overlap among females of different gentes would help distinguish between these hypotheses.

The parasitism of the redstart nest by a wagtail‐cuckoo likely represents a case of misdirected laying. We suspect that the wagtail‐cuckoo female had failed to find a suitable wagtail nest and subsequently laid her egg in the redstart nest instead. Indeed, the parasitic egg was markedly different from the host's blue eggs and quickly ejected, presumably by the host. Moreover, the redstart nest was already 6 days into incubation, making successful parasitism unlikely even if the egg had not been ejected. Such rare instances of misdirected laying may nonetheless provide opportunities for multiple parasitism when cuckoo females are effective at excluding females of the same gens but not females of different gentes.

Our observation highlights a largely unexplored aspect of cuckoo behavioural ecology: interactions among females belonging to the same or different gentes. Although our evidence is currently anecdotal, future studies investigating morphological and behavioural differences among females of different gentes, as well as their interactions and spatial overlap, may help clarify whether such factors influence patterns of multiple parasitism.

## Author Contributions

**Jinggang Zhang:** conceptualization (equal), data curation (equal), funding acquisition (equal), investigation (equal), methodology (equal), resources (equal), software (equal), writing – original draft (lead), writing – review and editing (equal). **Peter Santema:** writing – original draft (equal), writing – review and editing (equal). **Chenyang Zhao:** investigation (equal), writing – original draft (equal), writing – review and editing (equal). **Zixuan Lin:** investigation (equal), writing – original draft (equal), writing – review and editing (equal). **Jianqiang Li:** writing – original draft (equal), writing – review and editing (equal). **Wenhong Deng:** funding acquisition (equal), resources (equal), supervision (equal), writing – original draft (equal), writing – review and editing (equal). **Bart Kempenaers:** funding acquisition (equal), supervision (equal), writing – original draft (equal), writing – review and editing (equal).

## Funding

This study was supported by the Fundamental Research Funds for the Central Universities (310425209538 to J.Z.), the National Natural Science Foundation of China (32501383 to J.Z.; 32271559 to W.D.), and the Max Planck Society (to B.K.).

## Ethics Statement

Fieldwork was carried out with permission from Yongji Forestry Bureau, Jilin, China. Experimental procedures were conducted under licence from the Animal Management Committee at the College of Life Sciences, Beijing Normal University (permit no. CLS‐EAW‐2021‐003).

## Conflicts of Interest

The authors declare no conflicts of interest.

## Data Availability

All relevant data are described in the text or shown in the figure.
